# Effects of participatory organizational interventions on mental health and work performance: a systematic review and meta-analysis

**DOI:** 10.1093/joccuh/uiag024

**Published:** 2026-04-21

**Authors:** Asuka Sakuraya, Mako Iida, Kotaro Imamura, Kazuhiro Watanabe, Hiroki Asaoka, Emiko Ando, Akiomi Inoue, Reiko Inoue, Mai Iwanaga, Hisashi Eguchi, Yasumasa Otsuka, Yuka Kobayashi, Yu Komase, Kazuto Kuribayashi, Natsu Sasaki, Kanami Tsuno, Ayako Hino, Takeshi Ebara, Akihito Shimazu, Norito Kawakami, Akizumi Tsutsumi

**Affiliations:** Department of Digital Mental Health, Graduate School of Medicine, The University of Tokyo, Tokyo, Japan; Department of Mental Health, Graduate School of Medicine, The University of Tokyo, Tokyo, Japan; Department of Digital Mental Health, Graduate School of Medicine, The University of Tokyo, Tokyo, Japan; Department of Digital Mental Health, Graduate School of Medicine, The University of Tokyo, Tokyo, Japan; Department of Public Health, Kitasato University School of Medicine, Sagamihara, Japan; Department of Mental Health, Graduate School of Medicine, The University of Tokyo, Tokyo, Japan; Department of Psychiatry, Hori Mental Clinic, Fukushima, Japan; Institutional Research Center, University of Occupational and Environmental Health, Japan, Kitakyushu, Japan; Department of Public Health, Kitasato University School of Medicine, Sagamihara, Japan; Department of Community Mental Health & Law, National Center of Neurology and Psychiatry, National Institute of Mental Health, Tokyo, Japan; Department of Mental Health, Institute of Industrial Ecological Sciences, University of Occupational and Environmental Health, Japan, Kitakyushu, Japan; Institute of Human Sciences, University of Tsukuba, Tokyo, Japan; Department of Clinical Psychology, Faculty of Social Policy & Administration, Hosei University, Tokyo, Japan; Healthcare Development Division, Fujitsu Japan Limited, Kawasaki, Japan; Department of Psychiatric Nursing, Faculty of Chiba Nursing, Tokyo Healthcare University, Chiba, Japan; Department of Mental Health, Graduate School of Medicine, The University of Tokyo, Tokyo, Japan; School of Health Innovation, Kanagawa University of Human Services, Kawasaki, Japan; Department of Mental Health, Institute of Industrial Ecological Sciences, University of Occupational and Environmental Health, Japan, Kitakyushu, Japan; Department of Ergonomics, Institute of Industrial Ecological Sciences, University of Occupational and Environmental Health, Japan, Kitakyushu, Japan; Faculty of Policy Management, Keio University, Fujisawa, Japan; Department of Digital Mental Health, Graduate School of Medicine, The University of Tokyo, Tokyo, Japan; Department of Public Health, Kitasato University School of Medicine, Sagamihara, Japan

**Keywords:** organizational interventions, working environment, worker participation, well-being, productivity, worker involvement

## Abstract

**Objectives:**

Participatory organizational interventions (POIs) may improve workers’ mental health and work performance, but evidence from systematic reviews and meta-analyses of cluster-randomized controlled trials (cRCTs) remains limited. The meta-analysis has not been updated since it was published in 2015; a previous study focused on health care workers; and potential outcomes have not been adequately examined. This systematic review and meta-analysis aimed to investigate the effects of POIs on mental health and work performance among workers.

**Methods:**

The current study searched PubMed, EMBASE, PsycINFO, PsycARTICLES, and Japan Medical Abstracts Society for papers published up until November 11, 2022. Eligible studies were cRCTs assessing the effects of POIs on mental health or work performance. The risks of bias were evaluated by using the Cochrane risk-of-bias tool for cRCTs and meta-analysis was performed using a random effects model.

**Results:**

Fourteen cRCTs were included in the systematic review, and 8 cRCTs in the meta-analysis. The meta-analysis showed a nonsignificant beneficial effect on mental health conditions [standardized mean difference (SMD) = −0.04; 95% CI, −0.10 to 0.03], positive mental health (SMD = −0.004, 95% CI, −0.077 to 0.068), and work performance (SMD = 0.01; 95% CI, −0.10 to 0.13).

**Conclusions:**

This study did not confirm that POIs had a statistically significant effect on mental health or work performance. However, several studies that reported favorable results tended to emphasize active and structured participation, alignment with workers’ needs, and attention to organizational context. Further research is needed to identify the conditions under which the interventions are effective.

## Teaser

**Key points**
 

- **What is already known on this topic**:
  - Participatory organizational interventions (POIs) have the potential to improve workers’ mental health outcomes and work performance among workers.
  - A previous systematic review examined the effects of POIs on the mental health of health care workers (eg, occupational stress and burnout). However, their effects on the general working population, as well as on positive mental health and work performance, remain unclear.
- **What this study adds**:
  - The meta-analysis revealed no statistically significant effects of these interventions on any of the outcomes assessed among workers.
  - Studies reporting positive effects had characteristics such as greater emphasis on active and structured participation, alignment with workers’ needs, and attention to organizational context.
- **How this study might affect research, practice, or policy**:
  - For practice and policy, the findings suggest that flexible, context-sensitive interventions that actively engage workers may enhance both acceptability and effectiveness. While participatory approaches may not produce immediate or uniform effects, they can serve as valuable components in the ongoing process of workplace improvement.
  - Further research is needed to identify the conditions under which the interventions are effective (eg, levels of worker participation or alignment with workers’ needs).

## 1. Introduction

Work-related stress and poor psychosocial working conditions are major contributors to mental health problems and work performance among workers worldwide.^1^ According to the World Health Organization (WHO), mental health conditions are a leading cause of disability globally, and poor working environments can exacerbate these conditions.^1^^,^^2^ More than 12 billion days of lost productivity are attributable to depression and anxiety disorders each year, at an estimated cost of approximately US$1 trillion—highlighting the substantial economic burden of work-related mental health problems.^1^^,^^3^ To address these challenges, organizational interventions that target working conditions have been introduced to promote and sustain workers’ physical, mental, and social well-being across all occupations.^4^^,^^5^ Such interventions involve planned actions that primarily directly target working conditions with the aim of promoting and maintaining workers’ well-being.^5^ According to the International Organization for Standardization (ISO) 45003 guideline, worker participation is crucial throughout the intervention process.^6^ Participatory organizational interventions (POIs) typically involve workers participating in the steps of an intervention, such as action planning, implementing, evaluating, and reviewing the intervention.^5^ A previous report has indicated that worker participation enhances employees’ sense of control, fairness, justice, and support.^7^ In addition, involving workers helps optimize the fit of interventions to the organizational culture and context. Accordingly, it helps to ensure that organizational changes are better aligned with daily work practices.^7^ Therefore, worker involvement is regarded as a key component of organizational interventions. Furthermore, from a theoretical perspective, participatory approaches may be supported by occupational health models such as the job demands–resources model, which posits that enhancing job resources (eg, autonomy, participation in decision making, social support) can buffer job demands and promote employee well-being and performance.^8^ Given the growing emphasis on participatory workplace health and safety approaches, assessing their effectiveness and informing future implementation strategies is essential.

Multiple cluster-randomized controlled trials (cRCTs) have reported the impact of POIs. Some studies examined the effects on negative mental health outcomes, including depression or anxiety,^9^ as well as positive mental health outcomes, such as job satisfaction or well-being.^9^^,^^10^ Additionally, several studies investigated its effect on work performance, including job performance^11^ or absenteeism.^9^

However, systematic evidence on the effectiveness of POIs remains limited. Previous systematic reviews have examined the effects of organizational interventions on workers’ mental health and work-related outcomes, but the findings have been inconsistent. Several reviews have reported beneficial effects on outcomes such as depressive symptoms, burnout, or work-related outcomes, including absenteeism or sickness absence.^12–15^ In contrast, other recent reviews have found no significant effects on certain outcomes, such as burnout or psychological distress, or have reported mixed results across occupational groups.^16–18^ Importantly, most of these reviews combined participatory and nonparticipatory approaches, making it difficult to isolate the specific effects of worker participation. Moreover, a previous meta-analysis focusing on POIs examined only stress-related outcomes (eg, occupational stress and burnout) and did not find statistically significant effects; furthermore, the evidence included in that review was synthesized up to November 2013.^19^ In addition, existing evidence is largely restricted to health care workers, leaving other occupational groups (eg, office workers, manufacturing workers) underexplored. Outcomes beyond stress—such as positive mental health indicators and work performance—have also not been statistically synthesized. Furthermore, the methodological rigor of included studies, particularly with respect to study design and systematic risk-of-bias assessment, has varied across reviews. To address these limitations, an updated and methodologically rigorous synthesis focusing exclusively on cRCTs is warranted. cRCTs are particularly suitable for evaluating organizational-level interventions, as they reduce contamination between intervention and control groups and better reflect real-world workplace implementation. This study aimed to inform the development of evidence-based organizational policies and intervention programs, thereby contributing to healthier and more sustainable workplaces.

We systematically synthesized evidence from cRCTs evaluating POIs, compared with treatment as usual or no intervention (including waitlist control), on mental health outcomes and work performance among workers through a systematic review and meta-analysis.

## 2. Methods

### 2.1 Study design

This systematic review and meta-analysis for cRCT studies followed the Preferred Reporting Items for Systematic Reviews and Meta-Analyses (PRISMA 2020) guidelines.^20^ The study protocol was registered at the UMIN registry (registration number: UMIN000049453) and published elsewhere.^21^ Preliminary results were reported at the 98th Annual Meeting of the Japan Society for Occupational Health.^22^

### 2.2 Data sources and search strategy

The researchers conducted a systematic search of articles in November 2022 to retrieve articles published until November 11, 2022, across databases such as PubMed, EMBASE, PsycINFO, PsycARTICLES, and Japan Medical Abstracts Society. The search terms are published in Iida et al.^21^ In addition, articles found in the hand search were included for screening. The researchers defined the participants, interventions, comparisons, and outcomes (PICO) of the eligible studies as follows: (P) inclusion of all workers, (I) POI, (C) treatment as usual or no intervention (including waitlist control), and (O) mental health or work performance.^21^ In addition, eligible studies were those that (1) included POIs, (2) included participants who were working as of the baseline survey period, (3) assessed mental health or work performance outcomes, (4) used a cRCT design, (5) were published in English or Japanese, and (6) were published in peer-reviewed journals only (including advanced online publication).

### 2.3 Study selection process

Articles were organized using both EndNote-20 Library and Microsoft Excel files. M.Ii. removed duplicate entries. During the first screening phase, 15 investigators (Author 1, Author 2, Author 3, Author 4, Author 5, Author 6, Author 7, Author 8, Author 9, Author 10, Author 11, Author 12, Author 13, Author 15, and Author 16), together with 1 specialist staff member from an external contracting agency, independently assessed the article titles and abstracts in pairs based on the eligibility criteria. Next, the full texts of articles that passed the first screening were reviewed by pairs of investigators who conducted the first screening. The disagreements were solved by consensus of the authors. The reasons for exclusion were documented during the full-text review phase.

### 2.4 Data collection process

Data were extracted from the included studies mainly by 2 investigators (Author 1 and Author 2) and checked by other investigators, using a standardized data extraction form including the year of publication, the country in which the study was conducted, sample size, demographic characteristics of participants, the contents of the intervention, condition of the control group, outcome variables, and results of outcomes.

### 2.5 Risk-of-bias assessment

The study quality of each selected study was assessed by a total of 14 investigators (Author 1, Author 2, Author 3, Author 4, Author 5, Author 6, Author 7, Author 9, Author 10, Author 11, Author 12, Author 13, Author 15 and Author 16) independently by adopting the recently revised Cochrane risk-of-bias tool for cluster randomized trials (RoB 2 CRT).^23^ This tool consists of 6 domains: (1a) bias arising from the randomization process; (1b) bias arising from the timing of identification or recruitment of participants; (2) bias due to deviations from intended interventions; (3) bias due to missing outcome data; (4) bias in measurement of the outcome; and (5) bias in selection of the reported result. Each domain was assessed as: low risk of bias, some concerns, or high risk of bias. Any discrepancies in the risk-of-bias assessment were resolved by discussion and consensus among the authors.

Publication bias for meta-bias was evaluated using the Egger test as well as visually on a funnel plot.

### 2.6 Data synthesis and statistical methods

Meta-analyses were performed for the pooled effect on each outcome, including mental health outcomes and work performance. To account for differences in intervention effects across studies, the current study used a random-effects model using the method of DerSimonian and Laird.^24^ Because most of the outcomes collected from the included studies were continuous outcomes, the researchers synthesized them by calculating the standardized mean differences (SMDs) and their 95% confidence intervals (CIs). The results of the analysis considering clusters and the results of the analysis at the individual level were reported, but the latter accounted for the majority, so the researchers decided to integrate the differences in the means at the individual level. If categorical outcomes were reported, the researchers contacted the authors of the studies and asked if they could provide results for continuous outcomes. Further, when the information necessary for calculating SMDs and 95% CIs was lacking, the researchers also contacted the authors of the studies to request the necessary information. If there were multiple intervention arms, the intervention that applied to POIs was adopted for meta-analyses.^9^^,^^25^ For the mental health outcomes, various outcomes were reported, including depression, burnout, or sleep disorders. To investigate the effect on homogeneity, a subgroup meta-analysis was also conducted limited to stress-related outcomes such as depression, anxiety, stress, and negative affect. Heterogeneity between studies was assessed by using the Q statistic. Analysis was done with Stata version 18 (StataCorp LLC, College Station, TX, USA).

### 2.7 Change to protocol

The protocol did not include plans to conduct separate meta-analyses on mental health conditions and positive mental health.^21^ However, the included studies assessed heterogeneous mental health outcomes, including both mental health conditions (eg, depressive symptoms and exhaustion) and positive mental health indicators (eg, positive affect and work engagement). Therefore, to account for this heterogeneity, the researchers conducted separate meta-analyses for mental health conditions and positive mental health.

## 3. Results

### 3.1 Database searching

**Figure 1:**
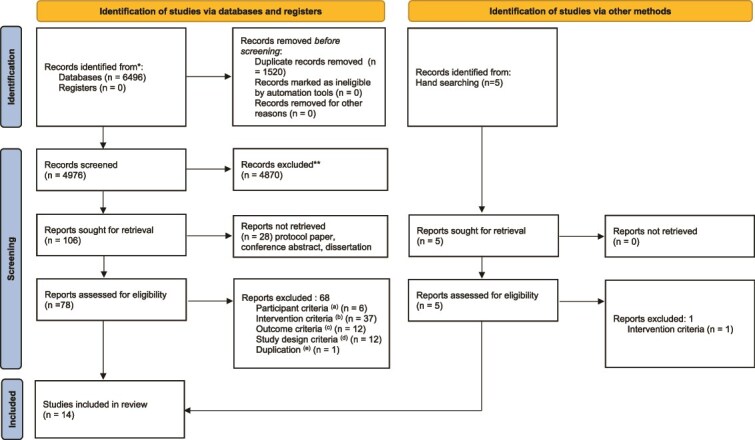
Flow diagram. a. Studies in which participants were not workers. b. Studies that did not involve participatory organizational interventions. c. Studies that did not assess mental health or work performance outcomes. d. Studies that did not use a cluster-randomized controlled trial design. e. Studies duplicated with other included studies. Source: Page MJ, et al. BMJ 2021;372:n71. doi: 10.1136/bmj.n71. This work is licensed under CC BY 4.0. To view a copy of this license, visit https://creativecommons.org/licenses/by/4.0/.

After removing duplicates, the current study included 4976 articles in the sifting phase. Next, 4870 articles were excluded, and 106 articles proceeded to full-text review. Finally, 14 studies^9–11^^,^^25–35^ were included in the systematic review (Figure 1). Among them, 4 were included from the hand search.^32–35^ For the meta-analysis, the current study used 8 studies. Six of the 14 studies were excluded from the meta-analysis because the necessary data to calculate SMDs and 95% CIs were unavailable despite attempts to contact the authors.

### 3.2 Study characteristics

The characteristics of the 14 studies are summarized in Table 1, including the PICO elements, country, and outcome results. Most of the studies were from Europe (Sweden, the United Kingdom, Denmark, Finland, the Netherlands, Norway, and Germany), but there were also some from the United States and Japan. Most of the research designs were 2-arm cRCTs, but there were also 3-arm cRCTs^9^ and 4-arm cRCTs,^25^ Among the populations included in the reviewed studies, most were employed in the health care sector or the public sector, whereas relatively few were from the private sector. For outcomes, 11 studies reported mental health conditions such as burnout or its subscales,^30^^,^^32^^,^^33^ depression or depressive symptoms,^9^^,^^27^ or anxiety.^9^ Five studies measured positive mental health such as job satisfaction or dissatisfaction,^9^^,^^26^^,^^28^^,^^30^ work engagement,^9^ or well-being.^9^^,^^10^ Seven studies measured work performance such as work ability,^10^ productivity,^10^ job performance,^11^ quality of medical care metrics,^31^ or absenteeism.^9^ Most interventions involved a process in which workers participated in multiple workshops, discussed the work environment, developed plans to improve it, and then implemented and reflected on the results. Although the specific intervention periods were not reported by some studies, for those that reported them, the period ranged from 1 hour^25^ to 25 months.^28^^,^^35^ More details of the interventions are provided in Table S1. Most of the studies utilized facilitators selected from among research group members, external practitioners or specialists, or key workplace personnel during the interventions. In addition, regarding intervention delivery, one study reported that 82% of clusters conducted at least one group catch-up session,^9^ and as for individual-level participation rates, reports of workshop participation rates ranged from about 60% to 100% (Table S1). However, many other studies did not clearly mention the participation rates. Favorable effects of the interventions were reported as improvements in mental health conditions such as reductions in burnout or its subscales (eg, emotional exhaustion and depersonalization),^30^^,^^32^^,^^33^ as well as improvements in work performance outcomes, such as increased job performance^11^ and reduced sickness absence.^35^ However, unfavorable effects were also observed, including deterioration in mental health conditions, such as increased emotional exhaustion,^33^ mental stress, and job dissatisfaction.^26^ In addition, 7 studies found nonsignificant effects for each outcome.

**Table 1:** Details of included cluster randomized controlled trials.

First author, year	Country	Population^a^	Sector	Intervention (contents)	Duration of intervention	Comparison	Outcome (scale of applicable)	Results on outcomes^b^
**Arapovic-Johansson et al, 2018** ^**29**^	Sweden	Employees at primary health care units Int: *n* = 56, *c* =1 Cont: *n* = 65, *c* = 2	Health care sector	The intervention was built on the Productivity Measurement and Enhancement System (ProMES). The main steps were as follows: (1) Formation of 1 or more design teams; (2) Identification of objectives; (3) Development of indicators; (4) Approval from management; (5) Development of contingencies; (6) Approval by management; (7) Development of feedback reports; (8) Conducting of feedback meetings; (9) Monitoring over time	Not specified (the last follow-up was conducted 12 mo after baseline)	1-h general feedback on selected results from the baseline questionnaire to the management The groups were also given written feedback material of results	Exhaustion (Oldenburg Burnout Inventory-OLBI) Problems with sleeping because of thinking about work (validated question from the Swedish project) Recovery (validated question from the Swedish project)	Exhaustion: 0 (no effect) Problems with sleeping because of thinking about work: 0 (no effect) Recovery: 0 (no effect)
**Edwardson et al, 2022** ^**9**^	United Kingdom	Employees at the council Int (no desk): *n* = 249, *c* = 27 Int (with desk): *n* = 240, *c* = 25 Cont: *n* = 267, *c* = 26	Public sector	The SMART Work and Life (SWAL) intervention is based on social cognitive theory, organizational development theory, habit theory, self-regulation theory, and relapse prevention theory. The intervention includes multifaceted strategies (organizational, environmental, individual, and group) that utilize the principles of the behavior change wheel and the associated COM-B (capability, opportunity, motivation, and behavior) approach One intervention group received SWAL only and the other group received SWAL and a height-adjustable desk	Not specified (the last follow-up was conducted 12 mo after baseline)	Usual practice	Anxiety (Hospital Anxiety and Depression Scale) Depression (Hospital Anxiety and Depression Scale) Stress (Perceived Stress Scale) Negative affect (Positive and Negative Affect Schedule) Occupational fatigue (Need for Recovery Scale) Sleep quality (Pittsburgh Sleep Quality Index) Sleep efficiency (an accelerometer) Positive affect (Positive and Negative Affect Schedule) Psychological well-being (World Health Organization-Five Well-Being Index (WHO-5)) Work engagement (Utrecht Work Engagement Scale) Job satisfaction (single item) Job performance (single item) Absenteeism (Self-report) Absenteeism (Organization records) Sickness presenteeism (Work Limitations Questionnaire)	No statistical testing was performed
**Eklöf and Hagberg, 2006** ^**25**^	Sweden	White-collar VDU workers Int 1 (individual feedback): *n* = 97, *c* = 9 Int 2 (feedback to supervisor): *n* = 106, *c* = 9 Int 3 (group feedback) *n* = 98, *c* = 9 Cont *n* = 95, *c* = 9	Private sector or public sector	Of the 3 interventions, Int 3 (group feedback), which is a participatory organizational intervention, is explained here During 1 session, experienced physiotherapists specialized in ergonomics provided normative information about computer ergonomics and psychosocial factors, and feedback information concerning the ergonomic and psychosocial situation among participants; also encouraged them to discuss implied problems and ideas for solutions. In the intervention of group feedback, 1-session feedback to the entire group with the supervisor present	1-h session (the last follow-up was conducted 6 mo after feedback)	No intervention (no feedback)	Emotional stress (a Mood Adjective Checklist)	Emotional stress: 0 (no effect)
**Gupta et al, 2018** ^**10**^	Denmark	Workers in Danish industrial workplaces Int: *n* = 193, *c* = 7 Cont: *n* = 222, *c* = 7	Private sector	Participatory physical and psychosocial workplace intervention (PIPPI) targets the individual, group, leader, and organizational levels of the workplace Group level: the workshops were attended by the team workers, their line manager, and a process facilitator who was either a member of the research group or an external consultant Individual level: workers were invited to voluntarily participate in an individual visual mapping talk with their line manager based on the tools from the visual mapping and action planning workshops Leader level: an ambassador workshop was conducted at each participating company Organizational level: the research group carried out an audit of the organizational systems, functions, and facilities. The audit results were fed back to local intervention steering committees in the individual organizations, and potential courses of action targeting the problems identified by the audit were discussed	Not specified (the last follow-up was conducted 12 mo after baseline)	Waitlist (based on protocol paper^36^)	Recovery (Need for Recovery Index) Mental health [3 items from the Medical Outcome Studies (MOS) Short-Form 36 (SF-36) Health Survey questionnaire and 2 items from the World Health Organization-Five Well-Being Index (WHO-5)] Well-being (WHO-5) Work ability (single work ability item) Productivity (single item)	Recovery: 0 (no effect) Work ability: 0 (no effect) Well-being: 0 (no effect) Mental health: 0 (no effect) Productivity: 0 (no effect)
**Haukka et al, 2010** ^**26**^	Finland	Workers in municipal kitchens of schools, kindergartens, and nursing homes Int: *n* = 263, *c* = 59 Cont: *n* = 241, *c* = 60	Public sector	The framework of the intervention (participatory ergonomics intervention) was based on the model developed at the Finnish Institute of Occupational Health. The active role of workers in collaboration with technical staff and management was emphasized. The researchers acted as consultants and trainers and facilitated the progress of the intervention process	The total duration varied from 11 to 14 mo (2-mo pre-implementation and 9 to 12 mo implementation phase)	Received no intervention	Mental stress during the past month (single item) Job dissatisfaction (single item)	Mental stress: − (unfavorable effect) Job dissatisfaction: − (unfavorable effect)
**Linzer et al, 2015** ^**30**^	United States (the upper Midwest and New York City)	Primary care clinicians Int: *n* = 83 Cont: *n* = 83^d^	Health care sector	Intervention sites chose a variety of methods to improve work-life and clinician outcomes such as improving communications, workflow, or quality improvement projects addressing clinician concerns	Approximately 12 mo (based on Linzer et al, 2017)^31^	Received no intervention (based on Linzer et al, 2017)^31^	The following outcomes were assessed by self-reported questionnaires: Burnout Stress Satisfaction	Burnout: + (favorable effect) Stress: 0 (no effect) Satisfaction: + (favorable effect)
**Linzer et al, 2017** ^**31**^	United States (the upper Midwest and New York City)	Clinicians and patients in clinics Int: *n* = 83 providers, *n* = 484 patients *c* = 17 clinics Cont: *n* = 82 providers, *n* = 402 patients *c* = 17 clinics	Health care sector	Same as Linzer et al, 2015^30^	Approximately 12 mo	Received no intervention	The following outcomes were evaluated by patients using quality and error metrics: Quality of care Medical errors	Quality of care: 0 (no effect) Medical errors: 0 (no effect)
**Tsutsumi et al, 2009** ^**11**^	Japan	Blue-collar workers employed in the company factory lines Int: *n* = 47, *c* = 6 Cont: *n* = 50, *c* = 5	Private sector	Team-based, problem-solving intervention based on active employee involvement, shared work-related goals, and action planning to improve the work environment for stress reduction. Training workshops for the facilitators, supervisor education program, set-up workshop, implementation of work environment improvements, and evaluation were included	Not specified The study was conducted over a period of approximately 1 year (from July 2005 to August 2006).	No organized activities were provided	Minor psychiatric morbidity [General Health Questionnaire (GHQ) 28-item version] Job performance [WHO Health and Work Performance Questionnaire (HPQ)]	Minor psychiatric morbidity (GHQ): 0 (no effect) Job performance (HPQ): + (favorable effect)
**Uchiyama et al, 2013** ^**27**^	Japan	Nurses in private, medium-sized general hospitals Int: *n* = 183, *c* = 11 Cont: *n* = 218, *c* = 13	Health care sector	A participatory program for improving psychosocial work environment was unit based, focused on active employee participation, and based on action planning to improve the work environment. All members in the intervention units were expected to participate in a series of activities designed to improve the work environment. Subchief nurses in each intervention unit were appointed as key persons to facilitate activities within their own unit	6 mo	Waitlist control	Depressive symptoms [Center for Epidemiologic Studies Depression Scale (CES-D)]	Depressive symptoms (CES-D): 0 (no effect)
**Le Blanc et al, 2007** ^**32**^ ^**c**^	The Netherlands	Staff members of oncology wards of general hospitals Int: *n* = 260, *c* = 9 Cont: *n* = 404, *c* = 20	Health care sector	A team-based burnout intervention program for oncology care providers (titled “Take Care!”). It was based on participatory action research approaches. During the action part, participants formed problem-solving teams that collectively designed, implemented, evaluated, and reformulated plans of action to cope with the most important stressors in their work situation	6 mo (the training program itself consisted of 6-monthly sessions of 3 h each)	Usual practice The wards signed a written agreement that they would refrain from participating in specialized training programs similar to the intervention	Emotional exhaustion and depersonalization (Maslach Burnout Inventory)	Emotional exhaustion:+ (favorable effect) Depersonalization:+ (favorable effect)
**Dahl-Jørgensen and Saksvik, 2005** ^**33**^ ^**c**^	Norway	Employees from the municipal administrative district Int: *n* = 84, *c* = 8 Cont: *n* = 100, *c* = 4 Employees in the shopping mall Int: *n* = 59, *c* = 17 Cont: *n* = 39, *c* = 13	Public sector or private sector	The aim of the intervention was to bring about changes in the factors perceived by employees as causes of stress at work and to bring about organizational changes at the unit level The method for uncovering the perceived causes of stress consisted of a 3-step strategy culminating in a meeting where employees and their supervisors engaged in group discussions or search conferences. Likewise, the organizational interventions were based on 3 theoretical traditions. The first focuses on participation, dialogue, and workplace democracy. The second deals with occupational stress and health research, with a primary emphasis on job redesign/environmental causes of illness, rather than on behavioral change at the individual level or on symptoms of ill health. The third research tradition is organizational learning theory, in which employees, through organizational inquiry, undergo a learning process during which they create qualitative improvements in the performance of organizational tasks and in the value system of the organization	1 year	Not specified	Emotional exhaustion and depersonalization (Maslach Burnout Inventory) Absenteeism (a self-reported measure of the number of sick days in the past 30 d) Somatic symptoms (a scoring system for subjective health complaints)	*Municipality* Emotional exhaustion: − (unfavorable effect) Depersonalization: 0 (no effect) Absenteeism: 0 (no effect) Somatic symptoms: 0 (no effect) *Shopping mall* Emotional exhaustion: 0 (no effect) Depersonalization: + (favorable effect) Absenteeism: 0 (no effect) Somatic symptoms: + (favorable effect)
**Montano et al, 2023** ^**34**^ ^**c**^	Germany	Health care workers in German health services institutions Int: *n* = 174, *c* = 22 Cont: *n* = 276, *c* = 47 (based on the protocol paper^37^)	Health care sector	The main aim of the intervention [“HALTgeben” (Higher Patient Satisfaction through Fair Working Conditions in Healthcare)] was based on the concept of work ability, which refers, in general, to the combination of the information about the workers’ health status and their appraisal of their own ability to meet the job demands. The intervention was conceived to help workers accomplish their work duties by considering how their individual characteristics and capacities may be aligned with the specific work and task processes. It was expected that this alignment would ultimately foster the perception of an increased work ability	Not specified The study was conducted over a period of approximately 2 years (from June 2019 to October 2021).	Waitlist control (based on protocol paper^37^)	The physical and mental work ability (Work Ability Index Questionnaire)	Physical work ability: 0 (no effect) Mental work ability: 0 (no effect)
**Framke et al, 2016** ^**35**^ ^**c**^	Denmark	All pedagogical leaders, nursery nurses, nursery nurse assistants, and other employees at pre-schools Int: *n* = 1760, *c* = 44 Cont: *n* = 1279, *c* = 34	Public sector	The intervention was designed as an open framework with no content requirements related to changing specific elements of the performance and organization of work. Employees’ participation in the development and implementation of workplace-specific intervention activities was pivotal. Pedagogical leaders in cooperation with employee representatives, shop stewards, and occupational health and safety representatives formed a steering group that managed the intervention. A working environment consultant was assigned to each workplace for the full intervention period. Intervention activities common for all workplaces were seminars and workshops for all steering groups on how to develop workplace-specific intervention activities, change management training, workplace culture, and evaluation	The intervention lasted 25 mo, counted from the date when workplaces were informed about group allocation (June 2011) until completion of the implementation phase (June 2013)	Not specified	The number of short-term sickness absence days per person-year (from the municipal sickness absence register)	Short-term sickness absence: + (favorable effect)
**Framke et al, 2016** ^**28**^	Denmark	Participants of Framke et al^35^ Employees who responded to both baseline and follow-up questionnaires^e^ Int: *n* = 944, *c* = 44 Cont: *n* = 616, *c* = 34	Public sector	Same as Framke et al^35^	Same as Framke et al^35^	Not specified	Exhaustion (1 item from the Major Depression Inventory) Sleep disturbances (1 item from the Major Depression Inventory) Job satisfaction (1 item)	Job satisfaction: 0 (no effect) Exhaustion: 0 (no effect) Sleep disturbances: 0 (no effect)

a *n*, number of participants (numbers assigned to each group); *c*, number of clusters (numbers assigned to each group); Int, intervention; Cont, control.

b +, favorable effect, –, unfavorable effect, 0, no effect.

c Included from hand search.

d The total number of clusters was 34, but the number of clusters in each group was not stated.

e Due to a high dropout rate after allocation, the final study sample was significantly reduced (Int *n* = 423, Cont *n* = 241).

### 3.3 Meta-analysis

**Figure 2:**
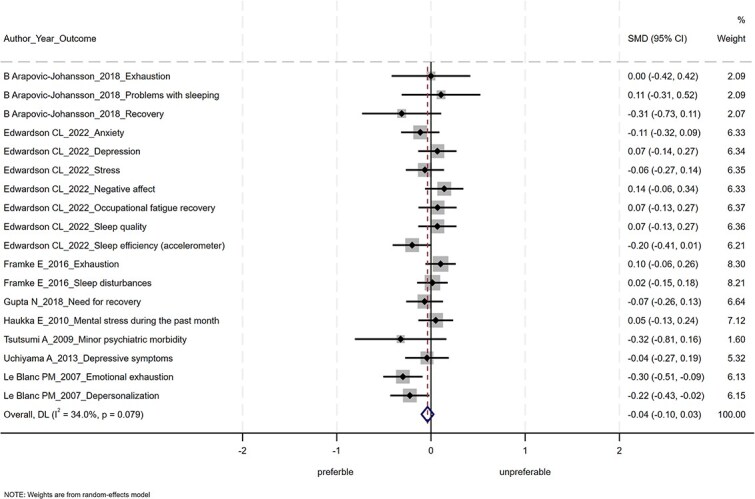
Forest plot of the meta-analysis for mental health conditions.

**Figure 3:**
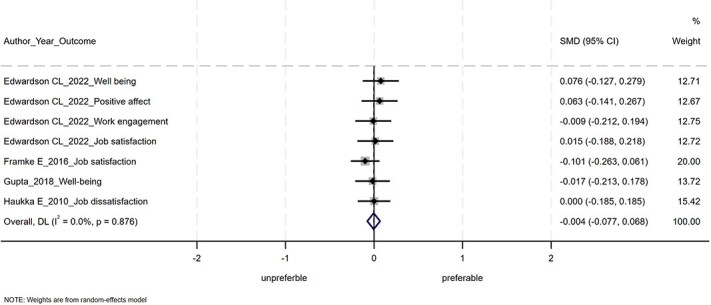
Forest plot of the meta-analysis for positive mental health.

**Figure 4:**
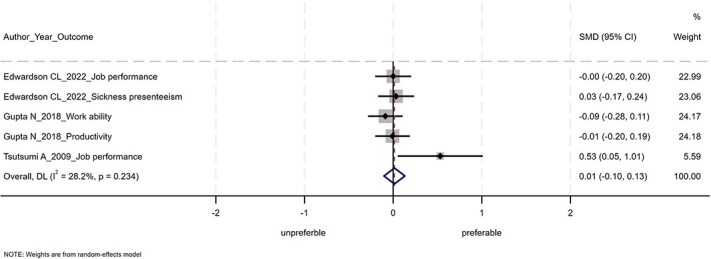
Forest plot of the meta-analysis for work performance.

The results of the meta-analysis are shown in Figures 2–4. Of the 14 studies, 6 were not included in the meta-analysis. This was because, despite contacting the authors, we were unable to obtain the information necessary to calculate the SMDs and 95% CIs. POIs had nonsignificant effects on improving mental health conditions (SMD = −0.04; 95% CI, −0.10 to 0.03), positive mental health (SMD = −0.004; 95% CI, −0.077 to 0.068), and work performance (SMD = 0.01; 95% CI, −0.10 to 0.13). The heterogeneity was not statistically significant for mental health conditions (Q = 25.76, df = 17, *P* = .08), for positive mental health (Q = 2.43, df = 6, *P* = .88), or for work performance (Q = 5.57, df = 4, *P* = .23).

The subgroup meta-analysis found no significant change in stress-related outcomes such as depression and anxiety (SMD = 0.00; 95% CI, −0.08 to 0.08).

### 3.4 Risk of bias

The results of risk-of-bias assessment by RoB 2 CRT are provided in Table 2. The overall risk of bias for all included studies was evaluated as high. Domains 1a and 1b were evaluated as Low risk or Some concerns in many studies; however, Domains 2 and 5 were evaluated as Some concerns. Additionally, Domain 4 was assessed as High risk in most studies.

**Table 2:** Summary of risk-of-bias assessment using the Cochrane risk-of-bias tool for cluster randomized trials (RoB 2 CRT) for the included studies.

First author, year	Domain 1a	Domain 1b	Domain 2 (effect of assignment to intervention)	Domain 2 (effect of adhering to intervention)	Domain 3	Domain 4	Domain 5	Overall
**Bozana Arapovic-Johansson, 2018**	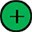	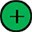	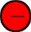		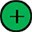	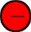	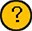	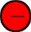
**Edwardson, 2022**	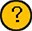	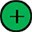	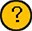		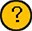	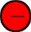	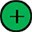	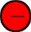
**Eklöf, 2006**	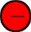	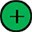	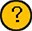		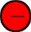	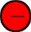	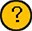	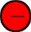
**Gupta, 2018**	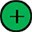	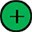	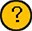		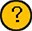	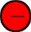	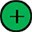	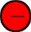
**Haukka, 2010**	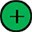	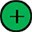	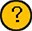		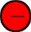	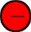	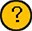	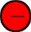
**Linzer, 2015**	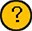	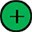	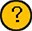		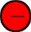	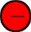	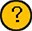	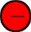
**Linzer, 2017**	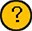	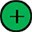		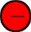	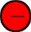	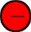	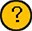	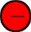
**Tsutsumi, 2009**	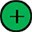	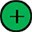	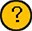		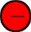	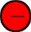	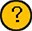	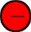
**Uchiyama, 2013**	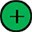	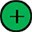	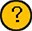		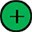	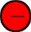	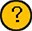	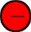
**Le Blanc, 2007**	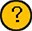	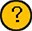	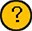		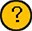	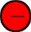	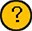	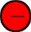
**Dahl-Jørgensen, 2005**	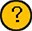	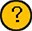	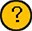		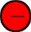	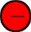	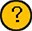	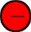
**Montano, 2023**	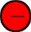	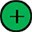	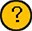		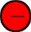	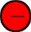	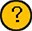	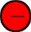
**Framke, 2016** ^**35**^	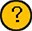	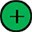	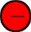		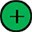	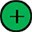	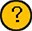	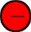
**Framke, 2016** ^**28**^	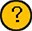	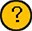		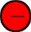	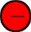	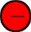	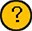	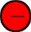

+, low risk of bias; ?, some concerns; −, high risk of bias. Domain 1a: Bias arising from the randomization process. Domain 1b: Bias arising from the identification or recruitment of participants into clusters. Domain 2: Bias due to deviations from intended intervention. Domain 3: Bias due to missing outcome data. Domain 4: Bias in measurement of the outcome. Domain 5: Bias in selection of the reported result. Note. Based on Sterne JAC, et al. BMJ. 2019;366:l4898. https://doi.org/10.1136/bmj.l4898.

### 3.5 Publication bias

Funnel plots of the included studies are shown in Figures S1–S3. For mental health conditions, the Egger test was nonsignificant. For positive mental health, the Egger test was significant (*P* = .01). Therefore, the researchers performed a sensitivity analysis on the 6 SMDs, excluding the one that was extremely low. The estimated pooled effect based on the 6 SMDs was also nonsignificant (SMD = 0.02; 95% CI, −0.06 to 0.10). The result of the Egger test was nonsignificant (*P* = .32). For work performance, the Egger test was significant (*P* = .02). A sensitivity analysis on the 4 SMDs, excluding the one that was extremely high, showed that the estimated pooled effect based on the 4 SMDs was also nonsignificant (SMD = −0.02; 95% CI, −0.12 to 0.08). The result of the Egger test was nonsignificant (*P* = .29).

## 4. Discussion

This systematic review and meta-analysis of cRCTs examined the effects of POIs on workers’ mental health and work performance. A total of 14 studies met the inclusion criteria. Outcomes included measures of negative mental health such as burnout, depression, and anxiety; positive mental health, including job satisfaction, well-being, and work engagement; and work performance indicators such as work ability and absenteeism. The meta-analysis revealed no statistically significant effects of the POIs on any of the outcome domains.

### 4.1 Implementation challenges and unintended consequences

In the present meta-analysis, POIs did not show statistically significant improvements in mental health conditions, positive mental health, or work performance. Even in subgroup analyses focusing specifically on stress-related outcomes such as depression and anxiety, no significant effects were observed. This lack of effect may partly reflect implementation challenges inherent to POIs. A study suggested that participation itself could impose additional demands on workers, as participatory processes—such as attending workshops, engaging in action planning, and implementing agreed changes—may constitute an additional work demand.^10^ Moreover, some studies reported adverse effects, including increased emotional exhaustion^33^ and elevated psychological stress.^26^ Although these adverse impacts were sometimes due to concurrent organizational reforms rather than the intervention itself,^26^ they may highlight the possibility of unintended side effects.^38^ Consistent with these findings, a previous implementation-focused study has shown that even organizationally anchored interventions may fail to improve stress-related outcomes with insufficient leader support, a low degree of role clarity, or concurrent organizational changes.^39^ Taken together, these findings suggest that POIs do not automatically improve mental health and may even be counterproductive if insufficiently resourced, poorly integrated into daily work, or implemented without adequate organizational support.

### 4.2 Contextual and process-related moderators of effectiveness

Despite the null overall effects, some individual studies reported positive results, indicating that POIs may be effective under certain conditions. Studies reporting favorable outcomes tended to place greater emphasis on worker participation based on participatory action research (PAR) approaches, treating it as an active and structured process during the intervention,^32^ alongside alignment with workers’ needs,^35^ and attention to organizational context.^35^ These findings are consistent with broader occupational health research suggesting that the interventions tend to be more effective when activities are aligned with workers’ perceptions of their work conditions or when the intervention is well integrated within the organizational context.^30^^,^^33^ Research in primary health care settings further indicates that leadership quality, relational climate, and opportunities for meaningful workplace dialogue may play a more decisive role in shaping psychosocial outcomes than the formal characteristics of the intervention itself.^40^ These findings suggest that POIs are highly context-dependent, and their effectiveness cannot be assumed without considering organizational readiness and perceived fit.

However, substantial limitations in process reporting were observed across the included studies. Few studies clearly reported 2 key aspects^5^: which steps—such as action planning, implementation, evaluation, and review—workers participated in during the intervention; and whether or not all workers participated in each step, or only selected worker representatives. Also, the period of the intervention was infrequently reported, limiting assessment of intervention fidelity. Evidence suggests that high worker participation rates in the planning workshops and high implementation rates of action plans are associated with stronger intervention effects.^41^ Therefore, a detailed understanding of which steps workers participated in and who participated at each stage is critical for accurately evaluating and improving the effectiveness of the intervention. Moreover, the readiness of workplaces to engage in POIs also plays a crucial role. Tsutsumi et al^11^ reported that interventions were less effective in workplaces characterized by low worker self-esteem, poor interpersonal relationships, and the presence of workers nearing retirement who resisted change. Consistent with this, previous research has highlighted that organizational conditions, including acceptance among workers, supervisors’ leadership, and proficiency in workplace dialogue, are key factors enabling meaningful improvements through POIs.^42^ Taken together, these findings underscore the importance of systematically evaluating both the fidelity of intervention processes and the organizational readiness in future studies to better clarify the conditions under which participatory approaches are most effective.

### 4.3 Methodological weakness

While the meta-analysis did not detect statistically significant effects, it is important to note that all included studies were judged to have a high overall risk of bias. Such methodological limitations may have reduced the reliability of the findings and obscured potential intervention effects. In particular, incomplete reporting of follow-up rates of clusters likely contributed to bias due to missing outcome data, and the predominant reliance on self-reported questionnaires introduced bias in the measurement of the outcome.

Beyond implementation challenges, the methodological limitations of conducting cRCTs in workplace settings has been emphasized. According to Rugulies et al,^43^ RCTs have several limitations to implementation due to the difficulties in fully randomizing or standardizing complex workplace interventions, as well as the influence of organizational and cultural contexts. For organizational-level interventions (cRCTs), such difficulties become even greater. Therefore, the challenges of accumulating evidence for randomization should also be recognized. To advance the evidence base, complementary methodological approaches may be needed. For example, stepped-wedge cRCTs can improve feasibility and ethical acceptability by allowing phased implementation while retaining randomization.^44^ To better explain contextual mechanisms, realist evaluation approaches can elucidate how, why, and under what organizational conditions POIs work,^45^ while mixed-methods designs can integrate quantitative outcome data with qualitative process and contextual information.^46^ Combining these approaches with cRCTs may therefore provide a more comprehensive evaluation of POIs.

### 4.4 Implications

#### 4.4.1 Implications for research

Future studies should incorporate systematic process evaluations, clearly report participation rates and roles at each intervention stage, and assess implementation fidelity. Greater transparency in reporting cluster-level follow-up and outcome measurement, along with rigorous risk-of-bias assessment, are essential. In addition, the use of complementary designs such as stepped-wedge trials, realist evaluation, and mixed-methods approaches may strengthen causal inference and contextual understanding.

#### 4.4.2 Implications for practice and policy

For practitioners and policymakers, the findings suggest that POIs are unlikely to be effective without adequate facilitation, strong managerial buy-in, and broad worker participation. Interventions should be tailored to the organizational context and workforce readiness, with sufficient time and resources allocated for meaningful participation. Consistent with WHO guidelines,^1^ POIs should be viewed as one component of a broader, integrated strategy for promoting mental health at work, rather than as standalone solutions.

### 4.5 Limitations

This study has several limitations. First, because the review was limited to studies published in English and Japanese, relevant studies in other languages may have been overlooked. Second, there may be unpublished studies—particularly those reporting negative findings—that were not captured. The current study assessed publication bias using a funnel plot and conducted the Egger test, which showed significant bias for positive mental health and work performance outcomes. Sensitivity analyses, including only studies that reported relevant SMDs or SEs were also performed, and these analyses showed no significant pooled effect of the interventions. Finally, although the current review aimed broadly to target all workers, most of the reviewed studies were conducted in the health care sector or the public sector, followed by the private sector. This limitation may reduce the generalizability of the current findings to the overall working population.

## 5. Conclusions

This systematic review and meta-analysis of cRCTs examined the effects of POIs on workers’ mental health and work performance. Overall, POIs did not produce statistically significant improvements across the examined outcomes. In contrast, several individual studies reported favorable results, emphasizing components such as active and structured participation, workers’ needs, and attention to organizational context. The heterogeneity of these interventions, together with inconsistencies in process reporting, emerged as a key challenge, making it difficult to evaluate their effectiveness at present. Further research should aim to establish standardized reporting criteria and identify the conditions under which POIs may be effective in improving mental health and work performance.

## Supplementary Material

Supplementary_materials_uiag024

## Data Availability

Data are available on reasonable request.
