# Supplementary material for: Effects of participatory organizational interventions on mental health and work performance: a systematic review and meta-analysis
Source: J Occup Health. 2026 Apr 21;68(1):uiag024. doi: 10.1093/joccuh/uiag024 (PMC13225270; doi:10.1093/joccuh/uiag024)
Supplement: Supplementary_materials_uiag024 [file supplementary_materials_uiag024.zip › Supplementary Table.docx]

| **Supplementary Table. Intervention characteristics** | | | |
| --- | --- | --- | --- |
| **First Author, Year** | **Intervention facilitators* *Includes those involved in facilitating, consulting on, or implementing the interventions.*** | **Participation rates of workshops** | **Managerial involvement for intervention** |
| Bozana Arapovic-Johansson, 2018 | 【External practitioners】  The consultant who was working with the intervention group is an independent, external practitioner, who is a certified user of ProMES and a highly experienced ProMES facilitator. He had worked with ProMES for 20 years and was one of the experts at the ProMES International Competence Centre. | Individual level  100%  An initial whole day workshop was attended by  all the employees in the intervention group. | - Management agrees with objectives and the　operationalization. - Management agrees with contingencies. |
| Charlotte L Edwardson, 2022 | 【Participants】  Workplace champions, who were council employees enrolled as participants in the study, were identified within each cluster and facilitated the interventions. | Cluster-level participation rates across both intervention arms (the SMART Work and Life (SWAL) only group and SWAL with a height adjustable desk group): 56% of clusters had both group catch-up sessions (82% having at least one). | The senior management team within each council allowed workplace champions protected time each month for facilitation of the interventions. |
| Mats Eklöf, 2006 | 【External specialists】  During 1 session, experienced physiotherapists specialized in ergonomics provided normative information about computer ergonomics and psychosocial factors, fed back information concerning the ergonomic and psychosocial situation among participants, and encouraged  them to discuss implied problems and ideas for solutions. | Individual level  96%  (88 of 92 participants) received group feedback. | 1-session feedback from physiotherapists to the entire group with the supervisor present. |
| Nidhi Gupta, 2018 | 【Research member or external specialists】  The workshops were attended by the team workers, their line manager, and a process facilitator who was either a member of the research group or an external consultant. | Individual level   - 61~75% of the workers in the group participated in the compulsory workshops. - 32% participated voluntary individual workshops. | Group level: The workshops were attended by the team workers, their line manager, and a process facilitator.  Organizational level: The audit results were fed back to local intervention steering committee (including upper and line management, local union representatives, and health and safety representatives), and potential courses of action targeting the problems identified by the audit were discussed. |
| Eija Haukka, 2010 | 【Researchers】  The researchers acted as consultants and trainers and facilitated the progress of the intervention process.  During the pre-implementation phase, researchers arranged two 5h workshops for workers in the intervention kitchens and gave interim support by visiting and phoning each kitchen.  During the implementation phase, researchers arranged six 3h workshops for workers in the intervention kitchens in each series and made extra visits on request. | Individual level  The average participation rate in the workshops was 73% (66% in the cities with no organizational reforms and 81% in the cities with reforms). | Food service managers and technical staff were invited to participate in the workshops. |
| Mark Linzer, 2015 | 【Research staff】  Research staff facilitated discussion among clinicians and provided guidance on the type or approach of interventions chosen. | Not specified | Not specified |
| Mark Linzer, 2017 | Same as Linzer et al., 2015. | Not specified | Quality improvement teams generally consisted of clinic managers and/or leaders, members of the department, and relevant providers and clinic staff (depending on the topic of the initiative). |
| Akizumi Tsutsumi, 2009 | 【Researchers and key persons of the study company】   - Before the baseline survey, researchers trained key persons of the study company who could take the initiative and facilitate participatory activities at their workplaces. - Before the set-up workshop, human resource personnel and the factory section chief served as facilitators in the supervisory education program. - In the set-up workshop, the facilitators led group discussions to assist the workers in listing activities for workplace improvements. - After the set-up workshop, facilitators supported and sustained employee autonomous activities during the implementation of work environment improvements. - Five and nine months after the set-up workshop, researchers held workplace observations and follow-up workshops, during which they gave necessary suggestions for further improvement and encouraged workers to sustain autonomous workplace improvement activities. | Not specified | - Before the intervention, several meetings were held with administrators and human relations personnel at the company to discuss the needs for improvement in work environment. - At the beginning of the set-up workshop, the factory manager declared that the factory would deal with mental health as a primary concern and participatory workplace improvements for stress reduction with the help of external experts. - The factory manager and facilitators participated in post-intervention interviews conducted by the researchers. |
| Ayako Uchiyama, 2013 | 【Researchers and Subchief nurses】   - The roles of the researchers were to observe, facilitate, and support the whole interventional process. - Subchief nurses in each intervention unit were appointed as key persons to facilitate activities within their own units. | Not specified | Subchief nurses in each intervention unit were appointed as key persons to facilitate activities within their own units. |
| Pascale M. Le Blanc, 2007 | 【External practitioners or specialists】  The program was developed in close collaboration with two experienced team counselors from an independent (i.e., unrelated to members of the research team) consultancy firm. | Individual level  Participation rates across the staff of each of the experimental wards varied from 80% to 100% (i.e., 80%–100% of the total sample size of these wards participated in all program sessions). | - Team counselors held extensive intake interviews with the management (e.g., head nurses, physicians, coordinators, and team leaders) of all wards. - The sessions were facilitated in several ways by the management. Meeting rooms were booked, and, as sessions often took place at the end of the day, catering arrangements for the participants were made. Moreover, in between the training sessions, the topics that were discussed during the latest session and the plans and agreements that were made were put as items on the agenda of the weekly work meetings of the respective experimental wards. |
| Carla Dahl-Jørgensen, 2005 | 【Without assistance from external expertise】   - The intervention was administered and implemented by the municipal units themselves without assistance from external expertise.   【Occupational health employees or researchers】   - Occupational health employees from the municipality implemented the intervention in some of the units. - At the shopping mall, the researchers assisted in the process of identifying problems, in creating ranking lists for discussions, and in administering the final group discussions. | Not specified | - After initial meetings between top-level managers and researchers about the intentions of the study, the project began. - The decision to implement the interventions was made at the administrative level in the organizations. - Managers and union representatives were asked to single out a factor they wanted to change, before determining a strategy for how this change could be accomplished (shopping mall). |
| Diego Montano, 2023 | 【External practitioners or specialists】  The intervention was conducted by consultants working in the field of work design and organizational development. | Not specified | - The intervention was motivated by personnel management following the invitation to participate in the study. - “Initiatives circles” were established which consisted of managing board executives, managers of the healthcare departments and representatives of the works council, quality management or human resources department. |
| Elisabeth Framke, Scand J Work Environ Health. 2016 | 【External practitioners or specialists】  The intervention was implemented by eight professional working environment consultants, with one consultant managing the implementation and securing that all workplaces received the same overall intervention. | Not specified | Not specified |
| Elisabeth Framke, BMC Public Health. 2016 | Same as Framke et al., Scand J Work Environ Health. 2016. | Same as Framke et al., Scand J Work Environ Health. 2016. | Same as Framke et al., Scand J Work Environ Health. 2016. |
