# Supplementary figures and images for: Effects of participatory organizational interventions on mental health and work performance: a systematic review and meta-analysis
Source: J Occup Health. 2026 Apr 21;68(1):uiag024. doi: 10.1093/joccuh/uiag024 (PMC13225270; doi:10.1093/joccuh/uiag024)

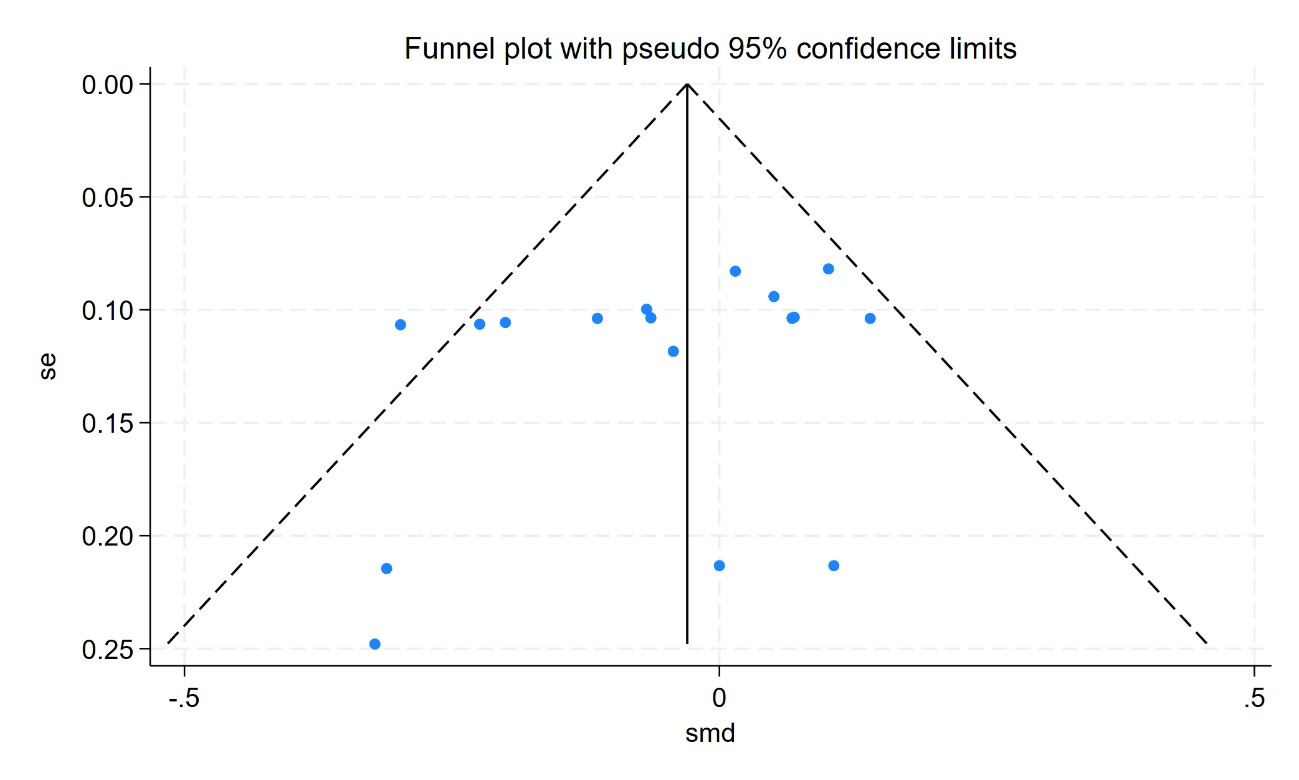

Supplement: Supplementary_materials_uiag024 [file supplementary_materials_uiag024.zip › Supplementary Figure1.jpg]

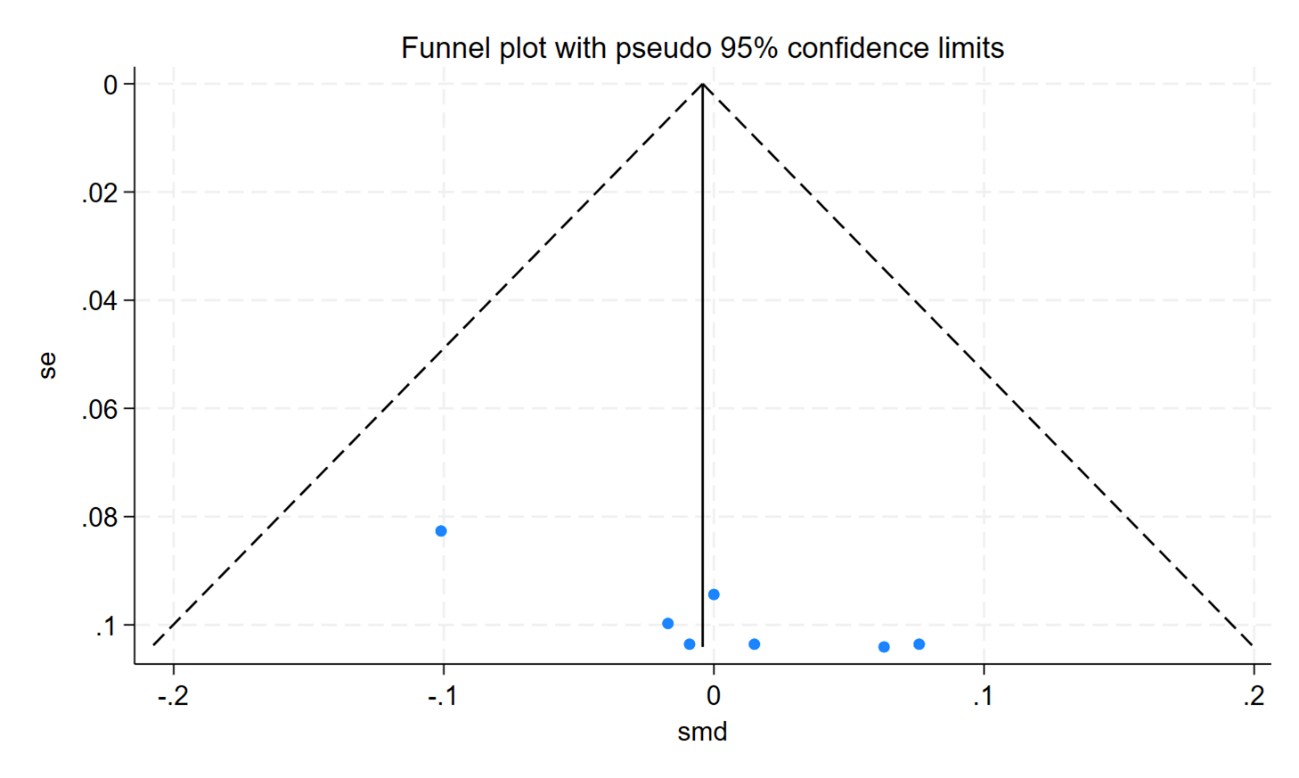

Supplement: Supplementary_materials_uiag024 [file supplementary_materials_uiag024.zip › Supplementary Figure2.jpg]

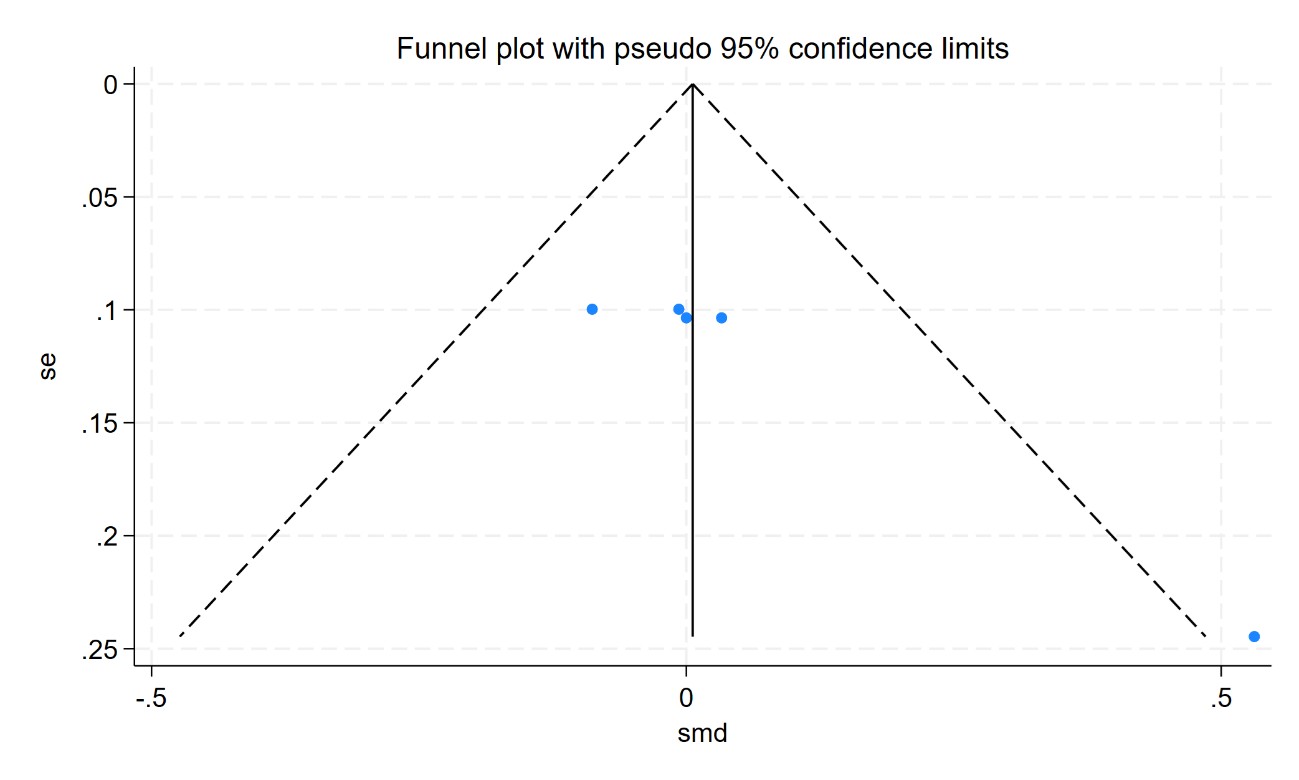

Supplement: Supplementary_materials_uiag024 [file supplementary_materials_uiag024.zip › Supplementary Figure3.jpg]
